# Troglitazone suppresses transforming growth factor beta-mediated fibrogenesis in retinal pigment epithelial cells

**Published:** 2008-01-18

**Authors:** Huey-Chuan Cheng, Tsung-Chuan Ho, Show-Li Chen, Huei-Yi Lai, Kuo-Fu Hong, Yeou-Ping Tsao

**Affiliations:** 1 Departments of Ophthalmology,; 2 Medical Research, Mackay Memorial Hospital, Taipei, Taiwan; 3 Department of Microbiology, School of Medicine, National Taiwan University, Taipei, Taiwan17117; 4 Department of Microbiology and Immunology, The National Defense Medical Center, Taipei, Taiwan

## Abstract

**Purpose:**

Transforming growth factor (TGF)-β2 induction of epithelial-mesenchymal transition of retinal pigment epithelium (RPE) cells has been implicated to be an important event during the development of proliferative vitreoretinopathy. The present study was conducted to examine whether troglitazone (TGZ) can inhibit TGFβ2-mediated fibrosis of RPE cells. The mechanism of the TGZ effect was also investigated by studying major TGFβ2-induced signaling including activation of Smad and p38 mitogen activated protein kinase (MAPK).

**Methods:**

Human RPE cells (ARPE-19) were exposed to various concentrations of TGZ in the presence of TGFβ2. The inhibitory effects of TGZ on collagen type I (COLI) and fibronectin (FN) expression induced by TGFβ2 was evaluated by reverse transcriptase-polymerase chain reaction. COLI synthesis was evaluated by the concentration of the C-terminal propeptide of COLI in the medium. The protein levels of FN and the phosphorylation of p38 MAPK and Smad2 and Smad3 were assessed by immunoblotting. TGZ inhibition of TGFβ2-promoted ARPE-19 cell migration was evaluated by an in vitro wound-healing assay. The influence of TGZ on cell viability was evaluated by the colorimetric conversion of 3-(4,5-dimethylthiazol- 2-yl)-2,5-diphenyltetrazolium bromide.

**Results:**

TGZ dose-dependently inhibited TGFβ2-induced COLI and FN overexpression at the levels of mRNA and protein manufacture. A dose-dependent TGZ inhibition was also apparent in TGFβ2-induced cell migration; cell viability was unaffected. TGFβ2 induced sequential phosphorylation of Smad2 and Smad3 and p38 MAPK. TGZ inhibited TGFβ2-induced early Smad2 and Smad3 and late Smad3 phosphorylation but had no influence on TGFβ2-induced p38 MAPK activation.

**Conclusions:**

TGZ pretreatment can significantly prevent TGFβ2-induced epithelial- mesenchymal transition of RPE cells, and retards cell migration. This may be achieved through the prevention of TGFβ2-induced Smad2 and Smad3 phosphorylation and subsequent nuclear accumulation. On the other hand, TGZ does not alter the levels of TGFβ2-induced p38 MAPK phosphorylation, the effect of TGZ is unlikely to be mediated by p38 MAPK signaling.

## Introduction

Retinal pigment epithelium (RPE) cells form a monolayer at the blood-retina barrier between the retina and choriocapillaries. Following retinal detachment, changes that frequently occur in RPE cells in the vitreous cavity and subretinal space include proliferation and production of extracellular matrix (ECM) components on the retina. This disease process is called proliferative vitreoretinopathy (PVR) [1,2]. The fibrous tissue on the detached retina ultimately reduces the flexibility of the detached retina [3] and becomes a major cause for failure of retinal reattachment surgery. Agents capable of preventing migration and fibrogenesis of RPE cells may be of great therapeutic value in improving the success rate of retinal reattachment surgery.

Transforming growth factor (TGF)-β is a potent fibrotic factor responsible for the synthesis of ECM. TGF-β plays a key role in pathogenesis of chronic fibroses, including kidney, liver, and lung [4-6]. Analysis of vitreous humor from patients who have experienced retinal detachments reveals levels of TGFβ2 that correlate with intraocular fibrosis and PVR severity [7]. In addition, RPE-mediated retinal contraction in an organ culture model can be reduced by the neutralizing antibody against TGFβ2; exogenous TGFβ2 can further stimulate RPE cell-mediated retinal contraction [8]. TGFβ2 may also function as an initiator to upregulate various PVR-inducing factors such as platelet-derived growth factor (PDGF) and connective tissue growth factor (CTGF) in the pathogenesis of PVR [9,10]. In cultured RPE cells, TGFβ2 induces the transformation of RPE to fibroblast-like cells [11], production of ECM such as collagen type I and fibronectin [9,12,13], and cell migration [13].

Phosphorylation of Smad3 and p38 mitogen-activated protein kinase (MAPK) are sequentially induced by TGFβ2, and both are important for mediating TGFβ2-induced fibrosis in ARPE-19 cells, a human RPE cell line that serves as an in vitro model [9,12,13]. The absence of fibrous tissue in the subretinal space in a mouse model of retinal detachment has been demonstrated in Smad3 null mice [9]. On the other hand, adenoviral gene transfer of dominant-negative (DN) p38MAPK to a mouse model of PVR has demonstrated the decreased ECM production in the subretinal space, consistent with a potential therapeutic efficacy via the inhibition of p38MAPK [13]. These results support the essential role of both signaling pathways in PVR. Interestingly, inhibition of p38 MAPK activity suppresses TGFβ2-induced ECM production of RPE cells but has no affect on TGFβ2-induced Smads2/3 phosphorylation [12,13]. However, it remains unclear whether p38 MAPK is a downstream effector of Smad cascade or is part of an independent signaling pathway contributing to fibrogenesis of RPE cells.

Thiazolidinediones (TZDs) such as troglitazone (TGZ) are a novel class of oral hypoglycemic drugs used to improve insulin resistance in non-insulin-dependent diabetes mellitus [14]. TZDs serve as ligands of peroxisome proliferator-activated receptor gamma (PPARγ), a ligand-dependent transcription factor that possesses pleiotropic effects; examples include regulation of adipogenesis, insulin sensitization, angiogenesis, and inflammation [15,16]. PPARγ ligands have the potential to suppress the fibrogenesis of hepatic stellate cells [17,18] and lung fibroblasts [19]. However, it is uncertain whether PPARγ ligands can suppress TGFβ2-mediated ECM production of RPE cells.

PPARγ ligands are capable of reducing fibrogenesis in several different types of cells [6,17-20]. This led us to investigate the influence of TGZ, which is a PPARγ agonist, on TGFβ2-mediated responses in RPE cells. Presently, we report that TGZ can efficiently inhibit production of ECM components and cell migration in TGFβ2-stimulated ARPE-19 cells. As well, TGZ can suppress TGFβ2-induced phosphorylation of Smad 2 and 3, providing a possible molecular mechanism to explain the TGZ inhibitory effect.

## Methods

### Cell Culture and treatment

Cells of the human RPE line ARPE-19 were obtained from the American Type Culture Collection (ATCC; Manassas, VA). ARPE-19 is an immortalized cell line that spontaneously arose from cultures of human RPE [21]. The cells were cultured in a humidified incubator at 37 °C in 5% CO_2_ in 10% fetal bovine serum-defined minimal essential medium (FBS-DMEM)-F12 medium supplemented with 100 U/mL penicillin G and 100 μg/mL streptomycin. When cultures achieved confluence, the spent medium was removed and replaced with fresh FBS-free medium. After 24 h of serum starvation, cells were treated with recombinant human TGFβ2 (R&D Systems, Minneapolis, MN) as previously described [12,13]. For inhibitor studies, cells were preincubated with SB203580 or TGZ (Calbiochem, San Diego, CA) for 1 h followed by TGFβ2 treatment. The inhibitors dissolved in DMSO (DMSO) were added to the cell culture (the final concentration of DMSO was less than 0.05%).

### Cell lysis, fractionation, and electrophoresis

ARPE-19 cells were scraped into lysis buffer (150 μL in each 35 mm well of the microtiter plate) containing 20 mM HEPES (pH 7.4), 1% sodium dodecyl sulfate (SDS), 150 mM NaCl, 1 mM EGTA, 5 mM β-glycerophosphate, 10 mM sodium pyrophosphate, 10 mM sodium fluoride, 100 μM sodium orthovanadate, 10 μg/mL leupeptin, and 10 μg/mL aprotinin. The lysate was incubated on ice for 15 min. Cell debris was removed by centrifugation (15,000 rpm, 15 min, 4 °C). For extraction of cytoplasmic and nuclear fractions, the NE-PER™ nuclear and cytoplasmic extraction kit (Pierce, Rockford, IL) was used according to the manufacturer’s instructions. Samples containing 20 μg of protein were analyzed by 12% SDS–PAGE and electrotransferred to polyvinylidene fluoride membranes (Immobilon-P; Millipore, Bedford, MA) and processed for western blotting.

### Western blot analysis

Samples were probed with anti-Active p38 pAb (Promega, Madison, WI), anti-phospho-Smad2 (S465/S467, Upstate Biotechnology, Lake Placid, NY), anti-phospho-Smad3 (S423/S425, R&D Systems, Minneapolis, MN), or anti-phospho-HSP27 (Ser78) antibody (Upstate Biotechnology) according to the manufacturer’s instructions, and then washed three times in Tris-buffered saline containing 0.1% Tween 20 (TBST). Antibody directed against p38/SAPK2 (Upstate Biotechnology), Smad2 (Abcam Ltd, Cambridge, UK), Smad3 (Zymed Laboratories, San Francisco, CA), or β-actin (Sigma-Aldrich, St. Louis, MO) were used to verify equal loading of protein. The blots were incubated with horseradish peroxidase-labeled anti-rabbit secondary antibody (Amersham Biosciences, Piscataway, NJ) diluted in TBST for 1 h and washed three times in TBST before visualization using an enhanced chemiluminescent technique. X-ray films were scanned on the Model GS-700 imaging densitometer (Bio-Rad Laboratories, Hercules, CA) and analyzed using Labworks 4.0 software (UVP, Upland, CA). For quantification, blots of at least three independent experiments were used.

### Measurement of the C-terminal propeptide of collagen type I

After treatment for 48 h, 20 μL of the conditioned medium was analyzed for collagen type I C-terminal peptide by an enzyme-linked immunosorbant assay, according to the manufacturer’s method (TaKaRa Biochemicals Co., Osaka, Japan).

### Semi-quantitative reverse transcriptase-polymerase chain reaction (RT–PCR)

Total RNA was extracted from ARPE-19 cells with TRIzol™ reagent (Invitrogen, Carlsbad, CA), according to the manufacturer’s protocol. Synthesis of cDNA was performed with 1 µg of total RNA at 50 °C for 50 min, using oligo (dT) primers and reverse transcriptase (Superscript III, Invitrogen). The amplification mixture (final volume 20 μl) contained 1 × Taq polymerase buffer, 0.2 mM dNTPs, 1.5 mM MgCl_2_, 1 μM primer pair, and 0.5 U of Taq DNA polymerase (Life Technologies, Inc., Gaithersburg, MD). cDNA was equalized in an 18–22 cycle amplification reaction with fibronectin primers 5`-tcgaggaggaaattccaatg-3` (forward) and 5`-ctcttcatgacgcttgtgga −3` (reverse), or collagen type I primers 5`-ggagggaatcactggtgcta −3` (forward) and 5`-agggggaaaaactgctttgt-3` (reverse) yielding a 300-bp product. The number of cycles for the primer set (denaturation for 20s at 94 °C; annealing for 30s and 59 °C; and polymerization for 40s at 72 °C) was chosen to be in the linear range of amplification.

### Cell migration assay

Cells (1 × 10^6^) were plated on 6-well culture plates (Corning, Corning, NY) in serum-free DMEM-F12. The cell layer was scratched with a pipette tip. The culture was then incubated with various reagents. Photographs of the wound were taken using a Nikon ECLIPSE TS100 microscope at various time points after monolayer wounding. Cell migration was evaluated by assaying the closure of a linear defect produced in a cell monolayer culture as previously reported [13].

### Cell viability

ARPE-19 cells were seeded in 10% FBS-DMEM-F12 medium in 96-well microculture plates (12,000 cells/well, Nunclon, Roskilde, Denmark) for 24 h. The culture medium was then replaced by serum-free DMEM-F12 medium for 16 h. At the time, the cell density before TGFβ2 or TGZ treatment was around 22,000 cells/well. Cells were received serum-free medium containing various concentrations of TGZ or were treated with 4 ng/ml TGFβ2 for 48 h. Cell viability was determined by the 3-(4,5-dimethylthiazol-2-yl)-2,5-diphenyltetrazolium bromide (MTT) assay, and confirmed by the trypan blue exclusion assay [22]. For performance of trypan blue exclusion assay, at the end of the cytotoxicity test, the cells were incubated with 0.05% trypan blue for 30 min.

### Statistical analysis

Data are expressed as mean ± standard deviation (SD) of three independent experiments, each done in triplicate (n=3–4 dishes). The Mann–Whitney *U* test was used to determine statistically significant differences. *P* values < 0.05 were considered significant.

## Results

### TGZ inhibits TGFβ2-induced fibronectin

**Figure 1:**
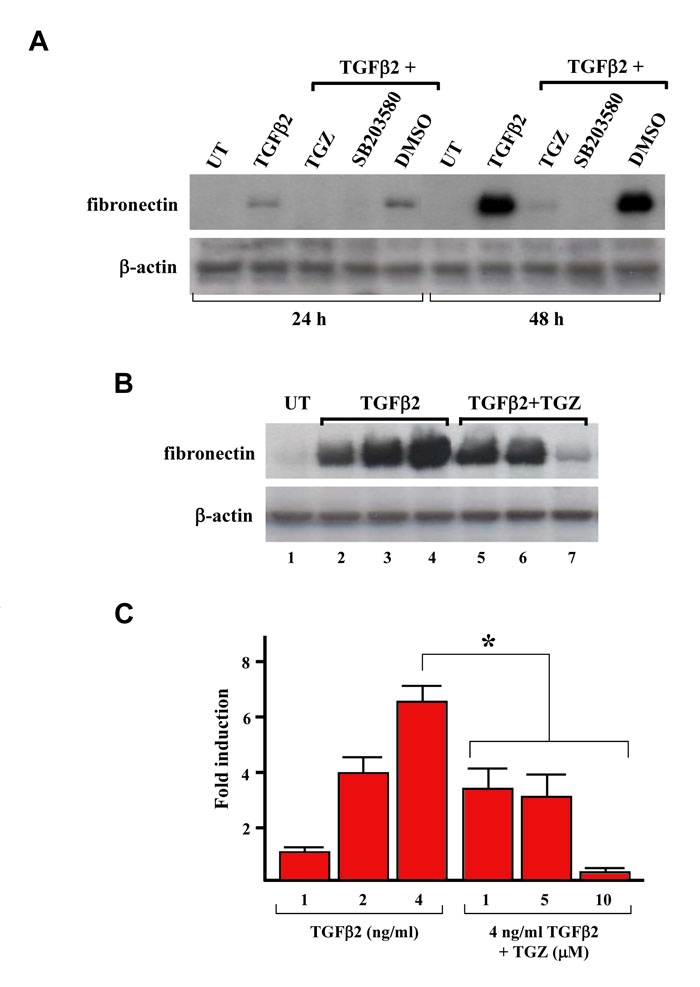
TGZ blocks TGFβ2-induced fibronectin expression in ARPE-19 cells.****A: Time-course study of the effect of TGZ on the TGFβ2-induced fibronectin expression. ARPE-19 cells were either left untreated (UT) or were treated with 4 ng/ml TGFβ2, or were pretreated with 10 μM TGZ, 10 μM SB203580 (p38 MAPK inhibitor), or DMSO for 1 h and then exposed to TGFβ2 for the indicated time periods. Immunoblot results are from a representative experiment performed in triplicate with β-actin as loading control. B: Dose study of the effect of TGZ on TGFβ2-induced fibronectin expression. ARPE-19 cells were treated with different doses of TGFβ2 (1, 2, 4 ng/ml; lanes 2–4) for 48 h or pretreated with different doses of TGZ (1, 5, 10 μM; lanes 5–7) for 1 h before 4 ng/ml TGFβ2 treatment for additional 48 h. Immunoblot results are from a representative experiment performed in triplicate with β-actin as loading control. **C:** After densitometric scans of triplicate blots, values for fibronectin were normalized to β-actin. *p<0.05 versus 4 ng/ml TGFβ2-treated cells.

Since fibronectin and collagen type I are the major ECM components of PVR tissue [13], we investigated whether TGZ could prevent TGFβ2-induced fibronectin protein in ARPE-19 cells. As shown in Figure 1A, western blot analysis revealed that exogenous TGFβ2 significantly increased fibronectin protein levels after treatment for 48 h as compared to 24 h treatment. Cells pretreated with 10 μM TGZ prevent the TGFβ2-mediated induction of fibronectin; at all time periods, the fibronectin level of the pretreated cells were similar as TGFβ2-untreated cells. DMSO pretreatment had no effect. p38 MAPK activation can be induced by TGFβ2 and is essential for TGFβ2-induced ECM production [12,13]. Our results also revealed that a similar inhibition effect was evident in cells pretreated with equal concentrations of a p38 MAPK inhibitor (SB203580) or TGZ.

Exposure of ARPE-19 cells to 1–4 ng/mL TGFβ2 for 48 h increased fibronectin protein levels in a dose-dependent manner (Figure 1B, lanes 2–4). To examine the TGZ dose effect on TGFβ2-induced fibronectin protein, cells were pretreated for 1 h with 1–10 μM TGZ before addition of 4 ng/ml TGFβ2 for a further 48 h. Immunoblotting results revealed that 1 and 5 μM TGZ could still temper suppress TGFβ2-induction of fibronectin protein (approximately 0.5-fold induction, similar to the induction produced by 2 ng/ml TGFβ2) (Figures 1B and 1C).

### TGZ inhibits TGFβ2-induced collagen type I

**Figure 2:**
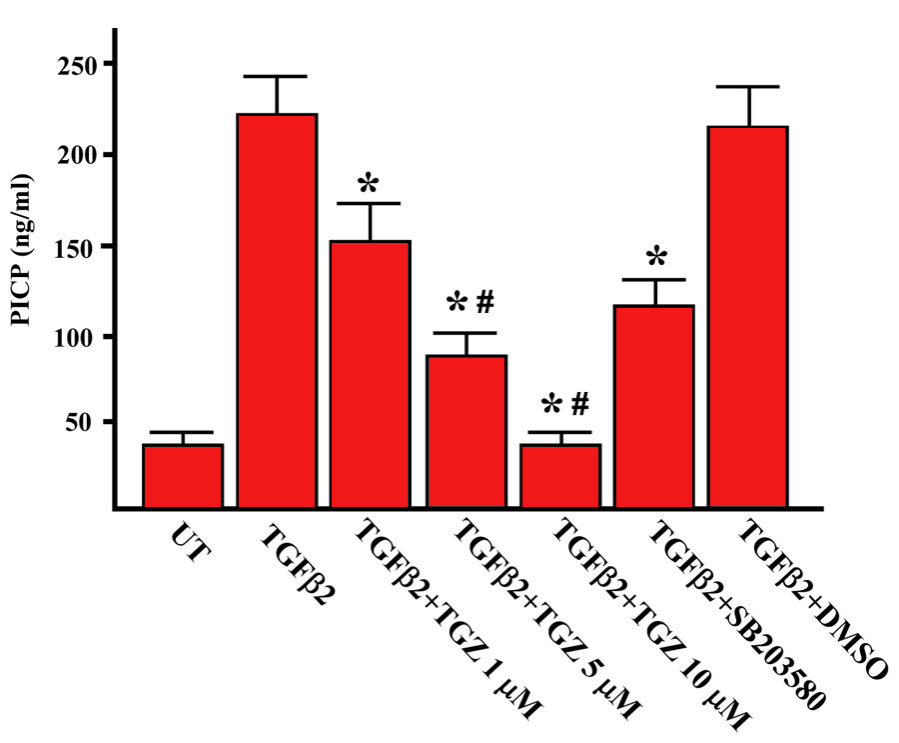
TGZ attenuates TGF-β2-induced collagen type I production in ARPE-19 cells. ARPE-19 cells were either left untreated or were treated with 4 ng/ml TGFβ2 for 48 h, or were pretreated with TGZ (1, 5, 10 μM), 10 μM SB203580, or DMSO for 1 h and then exposed to TGFβ2 for additional 48 h. The concentration of PICP (C-terminal propeptide of collagen type I) in the culture media under the various treatment conditions was measured by ELISA. Data represent the mean ± SD of results in four independent experiments. *p<0.01, compared with TGFβ2-treated cells. ^#^p<0.05, versus SB203580-pretreated cells.

To examine whether TGZ could prevent TGFβ2-induced collagen type I protein, we measured the concentration of the C-terminal propeptide of collagen type I (PICP) in the medium. As shown in Figure 2, exogenous TGFβ2 increased collagen type I protein levels, consistent with previous reports [12,13]. Cells pretreated for 1 h with 1–10 μM TGZ before the 48 h addition of 4 ng/ml TGFβ2 suppressed TGFβ2-induced collagen type I in a dose-dependent fusion. Pretreatment with DMSO had no such effect. In addition, 5 μM TGZ had an inhibitory effect exceeded that obtained following pretreatment with 10 μM SB203580.

### TGZ inhibits TGFβ2-induced collagen type I and fibronectin mRNA expression

**Figure 3:**
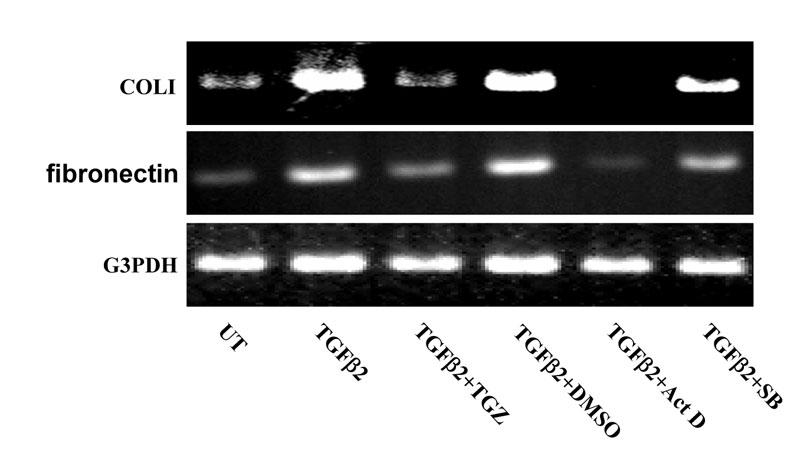
TGZ decreases TGFβ2-induced expression of fibronectin and collagen type I mRNA in ARPE-19 cells.****ARPE-19 cells were either left untreated or were treated with 4 ng/ml TGFβ2 for 24 h, or were pretreated with 10 μM TGZ, DMSO (0.05%), 10 ng/ml Actinomycin D, or 10 μM SB203580 for 1 h and then incubated with TGFβ2 for additional 24 h. Total RNA was extracted, and RT–PCR analysis for fibronectin and collagen type I (COLI) was performed. Glyceraldehyde-3-phosphate dehydrogenase (G3PDH) expression was examined for normalization purposes. Experiments were repeated twice and the results were reproducible.

Next, we investigated whether TGZ was capable of inhibition of TGFβ2-induced collagen type I and fibronectin mRNA expression. As shown in Figure 3, RT–PCR analysis demonstrated that ARPE-19 cells cultured in serum-free medium expressed a basal level of collagen type I and fibronectin genes; treatment with exogenous TGFβ2 for 24 h markedly induced expression of both mRNAs. Cells treated with TGFβ2 for 8 h also displayed an increased expression of both mRNAs but at levels less than following stimulation for 24 h (approximately threefold). The TGFβ2-mediated induction effect was completely blocked by actinomycin D pretreatment, suggesting that the increased mRNA expression was transcription dependent. Cells pretreated for 1 h with 10 μM TGZ before TGFβ2 treatment suppressed TGFβ2-induced collagen type I and fibronectin mRNA to basal levels that were comparable to untreated cells. Pretreatment with DMSO had no such effect. Similarly, 10 μM SB203580 pretreatment partially prevented TGFβ2-induced collagen type I mRNA expression (compare Figure 2 with Figure 3).

**Figure 4:**
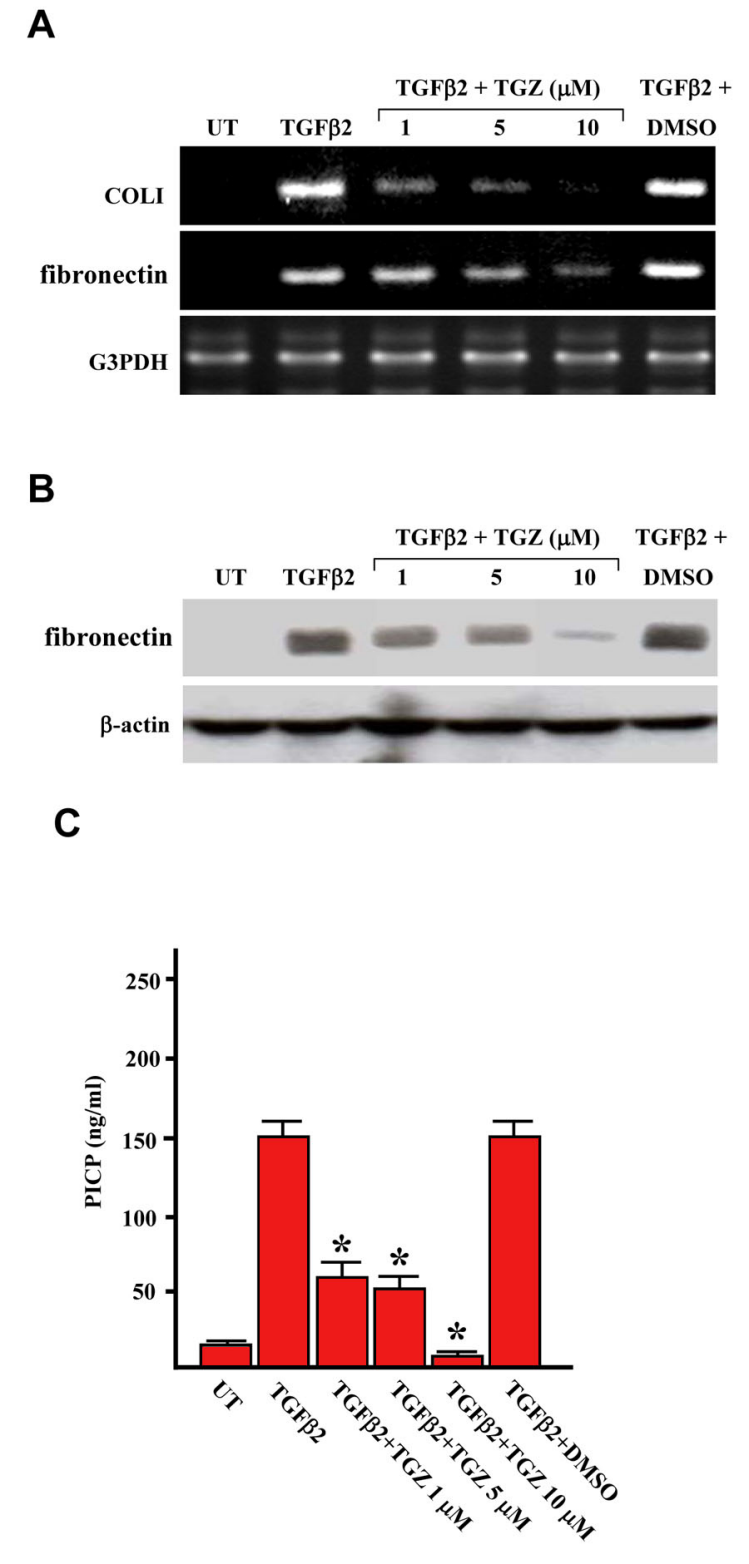
TGZ suppresses TGFβ2-induced expression of fibronectin and collagen type I in subconfluent ARPE-19 cells.****ARPE-19 cells at subconfluent density of 5 X 10^5^ cells per well in six-well plates were either left untreated (UT) or were treated with 4 ng/ml TGFβ2, or were pretreated with different doses of TGZ (1, 5, 10 μM; 1 h) or DMSO (0.05%, 1 h) and then incubated with TGFβ2. The expressions of fibronectin and collagen type I were assayed by RT–PCR analysis (A), western blot analysis (B), and ELISA (C) as Figures 1–3 described. *p<0.05 versus TGFβ2-treated cells. Experiments were repeated twice and the results were reproducible.

Next, we investigated whether TGZ could prevent TGFβ2-induced expression of fibronectin and collagen type I in subconfluent ARPE-19 cells. As shown in Figure 4A, RT–PCR analysis revealed that both mRNAs were markedly upregulated by TGFβ2 treatment for 24 h and the TGFβ2-mediated induction effect was blocked by TGZ pretreatment followed a dose-dependent manner. Pretreatment of subconfluent ARPE-19 cells with TGZ (1–10 μM, 1h) also dose-dependently suppressed TGFβ2-induced fibronectin and collagen type I proteins as assayed by western blot analysis and ELISA (PICP), respectively (Figure 4B and 4C). DMSO pretreatment had no effect. Collectively, our results revealed that TGZ suppresses TGFβ2-mediated fibrogenesis in ARPE-19 cells grown to either confluence or subconfluence.

### TGZ inhibits TGFβ2-induced cell migration

**Figure 5:**
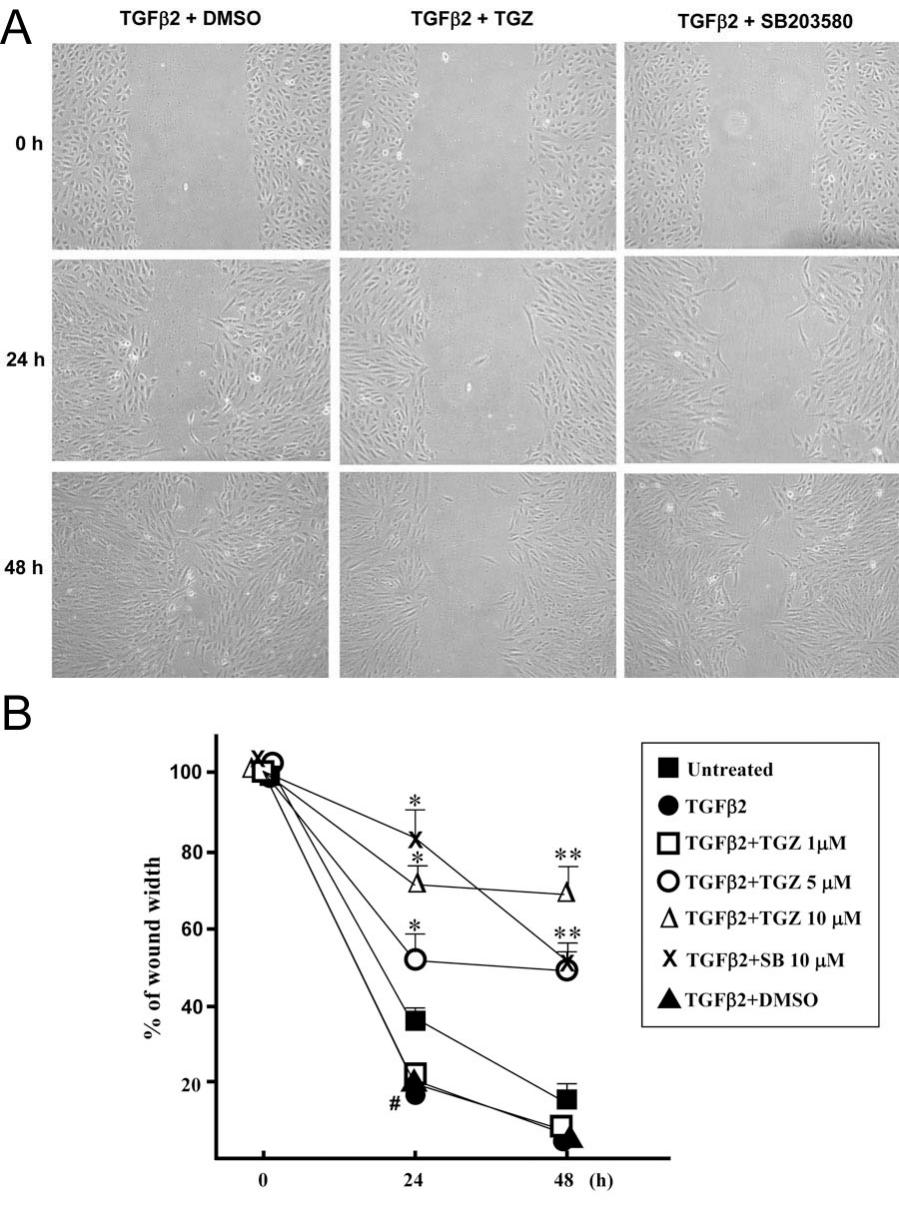
TGZ reduces TGFβ2-promoted healing of wounds in ARPE-19 cell monlayers.****Cell monolayers were pretreated with vehicle (DMSO), TGZ (1, 5, 10 μM), or 10 μM SB203580 for 1 h and then treated with 4 ng/ml TGFβ2 and wounded with a P-200 pipette tip immediately after 0 h. Wells were photographed at 0, 24, and 48 h adjacent to a reference line scraped on the bottom of the plate. A: Panels show migration of cells pretreated with either DMSO, 10 μM TGZ, or 10 μM SB202190 before treatment with TGF-β2 for 0, 24, and 48 h as indicated. B: Percent of remaining wound width in monolayer cell sheet. The data show the percent remaining wound closure in each culture condition. For this value, the width was measured at three different locations in the wound and the mean value was compared to the width of the original closure (0 h). All experiments were in triplicate. Bars represent the mean ± SD ^#^p<0.01 versus 24 h untreated cells and *p<0.05 versus 24 h TGFβ2-treated cells. **p<0.05 versus 48 h TGFβ2-treated cells.

TGFβ2-mediated promotion of RPE cell migration has been implicated in the development of PVR [23]. We examined this in more detail using an in vitro wound healing assay in which ARPE-19 cell migration was quantified by the width of remaining defect. As shown in Figure 5B, addition of TGFβ2 significantly enhanced the cell migration (compared untreated cells with TGFβ2-treated cells at 24 h post-wounding; p<0.01). Cells pretreated with 5 and 10 μM TGZ suppressed TGFβ2-promoted cell migration in all the time periods studied (approximately 50 and 72% of the remaining defect as compared to the original defect), but 1 μM TGZ or DMSO had no inhibitory effect. Figure 5A shows representative photographs of cells migrating into scratch wounds. Our results also revealed that a similar inhibition effect was obtained from cells pretreated with 10 μM SB203580 or 5 μM TGZ before TGFβ2 treatment for 48 h (**p<0.05 versus 48 h TGFβ2-treated cells).

**Figure 6:**
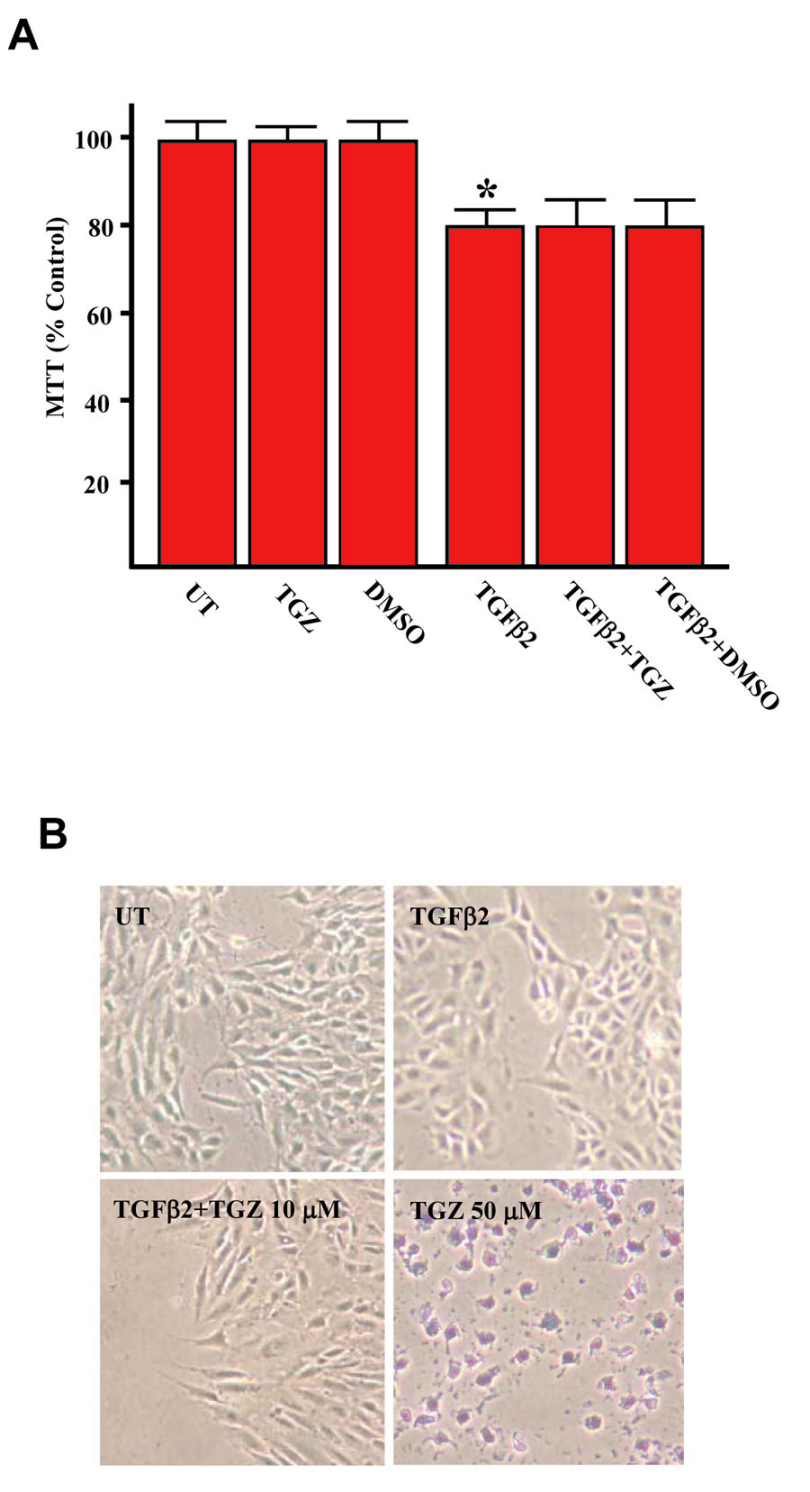
TGZ at 10 μM has no effect on ARPE-19 cell proliferation either in the presence or absence of TGFβ2.****ARPE-19 cells were either left untreated or treated with 10 μM TGZ, 0.05% DMSO, or 4 ng/ml TGFβ2, or were treated with TGFβ2 combining with TGZ or DMSO for 48 h as indicated. Cell viability was estimated by MTT assay (A) or trypan blue exclusion assay (B). TGZ at 50 μM is cytotoxic and used as positive control of trypan blue exclusion assay. Bars represent the mean ± SD *p<0.05 versus untreated cells.

To confirm that the effect of TGZ on wound gap was not a result of TGZ cytotoxicity on ARPE-19 cells, cells were exposed to increasing concentrations of TGZ (1–10 μM) in serum-free medium for 48 h, and cell viability was examined using an MTT assay. As shown in Figure 6A, TGZ did not affect cell viability even in cells treated with 10 μM TGZ, although proliferation of ARPE-19 cells was evident; the latter is consistent with another study [13]. However, 10 μM TGZ showed no further inhibitory effect on cell proliferation in the presence of TGFβ2. To further confirm that TGZ at 10 μM does not affect RPE survival at the region of wound healing, cell viability was evaluated using trypan blue exclusion assay. Results revealed that 10 μM TGZ did not affect cell survival while 50 μM TGZ treatment caused massive cell death and trypan blue staining (Figure 6B).

### The TGZ inhibition effect is independent to the activation of PPARγ

**Figure 7:**
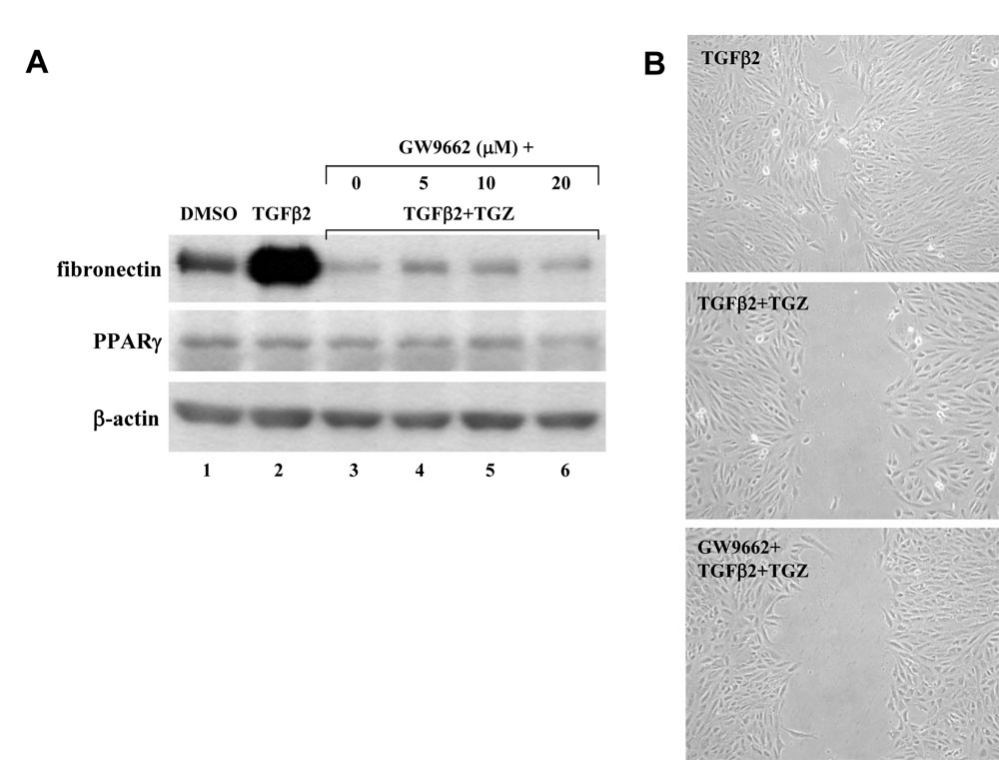
The TGZ inhibition effect is independent to PPARγ activation.** A:** ARPE-19 cells were pretreated with different doses of GW9662 (5–20 μM, 2 h) before TGZ combing with TGFβ2 treatment for additional 48 h. Immunoblot results are from a representative experiment with β-actin as loading control. **B:** ARPE-19 cell monolayers were either treated with TGFβ2 or pretreated with 10 μM GW9662 before TGZ combing with TGFβ2 treatment and wounded with a P-200 pipette tip immediately. Wells were photographed at 48 h adjacent to a reference line scraped on the bottom of the plate.

To investigate whether TGZ acts through the activation of its receptor PPARγ to suppress TGFβ2-mediated reactions, ARPE-19 cells were pretreatment for 2 h with 5–20 μM GW9662, a specific PPARγ antagonist, and then assayed for PPARγ levels, fibronectin levels and cell migration in the presence of 10 μM TGZ combining with TGFβ2. As showed in Figure 7A, western blots showed that there was no change in the PPARγ protein levels with exposure to TGZ in the presence of TGFβ2. In addition, GW9662 did not reverse the inhibition of TGFβ2-induced fibronectin production (Figure 7A). in vitro wound healing assay also revealed that TGZ suppressed TGFβ2-promoted cell migration can not be reversed by GW9662 (Figure 7B, compared TGFβ2+TGZ with GW9662+TGFβ2+TGZ-treated cells at 48 h post-wounding). Therefore, the results led us to suggest that TGZ-mediated inhibitions are through a PPARγ-independent mechanism.

### TGZ does not affect the TGFβ2-induced p38 MAPK phosphorylation

**Figure 8:**
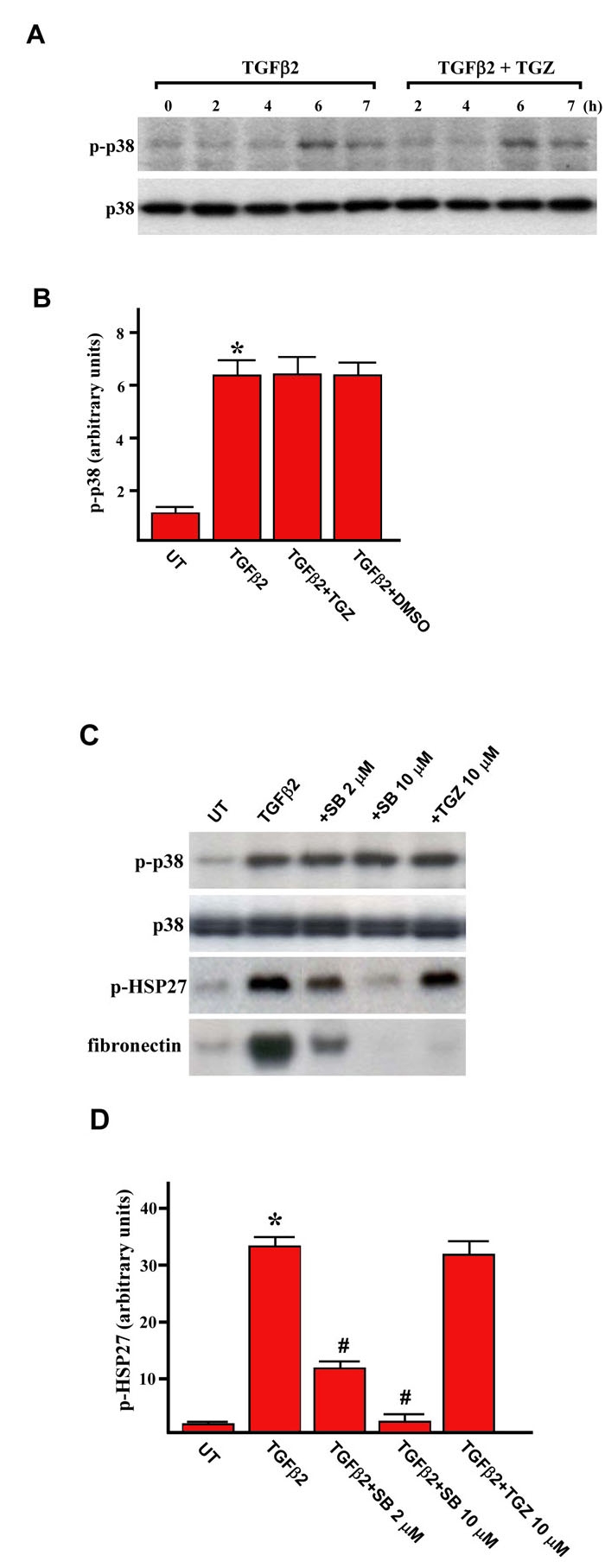
The effect of TGZ on TGFβ2-induced p38 activation.****A: ARPE-19 cells were treated with TGFβ2 alone or were pretreated with 10 μM TGZ for 1 h and then stimulated with TGFβ2 for the indicated time periods. Cells were harvested and subjected to western blot analysis with phosphospecific antibodies of p38 MAPK. Equal protein loading was confirmed by the reprobing of membranes with total p38 MAPK antibody. B: After densitometric scans of triplicate blots, values for p-p38 MAPK (after the induction for 6 h) were normalized to total p38 MAPK. *p<0.05 versus untreated cells. C: ARPE-19 cells were treated with TGFβ2 alone or were pretreated for 1 h with the SB203580 or TGZ at the indicated concentrations and then stimulated with TGFβ2 for an additional 6 h. Cells were harvested and subjected to western blot analysis with antibodies against phosphorylated forms of p38 MAPK and HSP27. Loading equality was confirmed with antibodies against total p38 MAPK. D: After densitometric scans of triplicate blots, values for p-HSP27 were normalized to total p38 MAPK. *p<0.05 versus untreated cells. ^#^p<0.05 versus TGFβ2-treated cells.

We further investigated whether TGZ affected TGFβ2-induced p38 MAPK signaling to suppress TGFβ2-mediated responses in ARPE-19 cells. Cells were treated with TGFβ2 or pretreated for 1 h with 10 μM TGZ before TGFβ2 treatment for various time periods. Subsequently, the phosphorylation status of the p38 MAPK was detected by immunoblotting. As shown in Figure 8A and 8B, TGFβ2 stimulation caused phosphorylation of p38 MAPK, which was transiently upregulated at 6 h as previously reported [13]. However, TGFβ2 still caused a similar p38 MAPK phosphorylation in TGZ-pretreated cells in all the time periods studied, suggesting TGZ can non inhibit the p38 MAPK phosphorylation. In addition, DMSO had no effect on basal and TGFβ2-induced p38 MAPK phosphorylation (data not shown).

To further ensure that TGZ dose not affects p38 MAPK signaling, we examined the levels of phosphorylation of HSP27, a documented substrate of p38 MAPK signaling in ARPE-19 cells [24]. Results showed that TGZ, at the concentration that can attenuate fibronectin expression (Figure 8C), does not change the levels of HSP27 phosphorylation induced by TGFβ2 treatment (Figure 8C and 8D), whereas SB203580 dose-dependently inhibited HSP27 phosphorylation. These suggest that TGZ inhibit TGFβ2-induced fibrogenesis through a p38 MAPK-independent mechanism.

### TGZ suppresses TGFβ2-induced Smad phosphorylation

**Figure 9:**
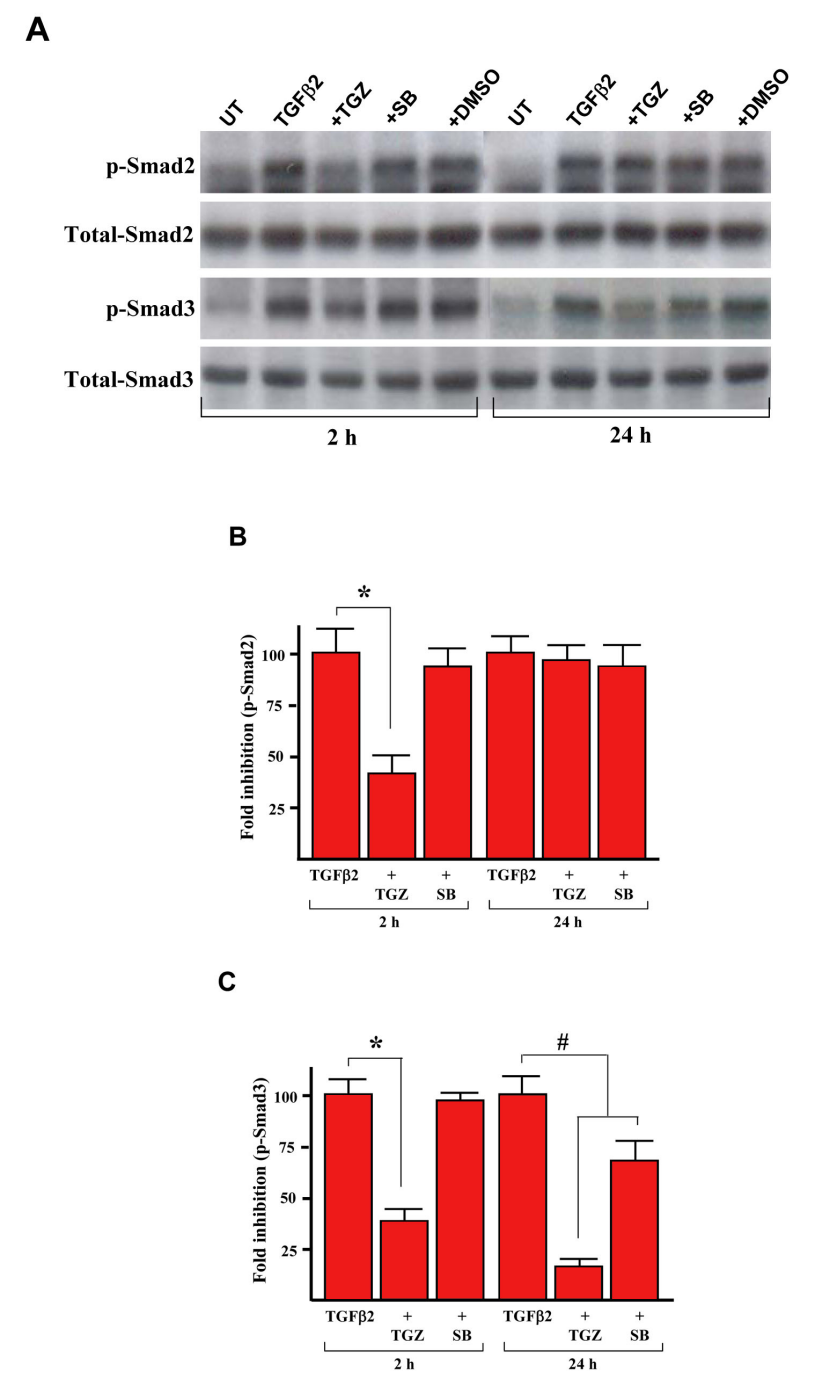
TGZ time-dependently suppresses TGFβ2-induced Smad phosphorylation. A: ARPE-19 cells were treated with 4 ng/ml TGFβ2 alone or were pretreated with 10 μM TGZ, 10 μM SB203580, or DMSO for 1 h and then stimulated with TGFβ2 for the indicated time periods. Cells were harvested and subjected to western blot analysis with phosphospecific antibodies of Smad2 and Smad3. Equal protein loading was confirmed by the reprobing of membranes with total Smad2 and Smad3 antibody as loading control. B and C: After densitometric scans of triplicate blots, values for phospho-Smad2 and Smad3 were normalized to total Smad2 and Smad3, respectively. *p<0.02 versus TGFβ2-treated cells. ^#^p<0.05 versus TGFβ2-treated cells

Since Smad signaling has been implicated in TGFβ2-dependent induction of ECM components [9], we examined whether the TGZ could affect C-terminal phosphorylation of Smad 2 and 3 by western blot analysis. As shown in Figure 9A, cell exposure to TGFβ2 induced phosphorylation of both Smad species not only after treatment for 2 h, but also was sustained for 24 h, as in previous studies [9,13]. SB203580 had no significant effect on the phosphorylation of Smad2 in all the time periods studied but partially prevented phosphorylation of Smad3 after TGFβ2 stimulation for 24 h (65 ± 12% lower than TGFβ2-treated cells; Figure 9C). Exposure of cells to 10 μM TGZ partially suppressed TGFβ2-induced Smad2 phosphorylation (36 ± 8% versus TGFβ2 treatment for 2h) but had no effect on the levels of Smad2 phosphorylation stimulated by TGFβ2 for 24 h (Figures 9A and 9B). Notably, TGZ suppressed the levels of Smad3 phosphorylation stimulated by TGFβ2 for either 2 h (41 ± 4%) or 24 h (15 ± 3%) relative to TGFβ2-treated cells (Figures 9A and 9C).

**Figure 10:**
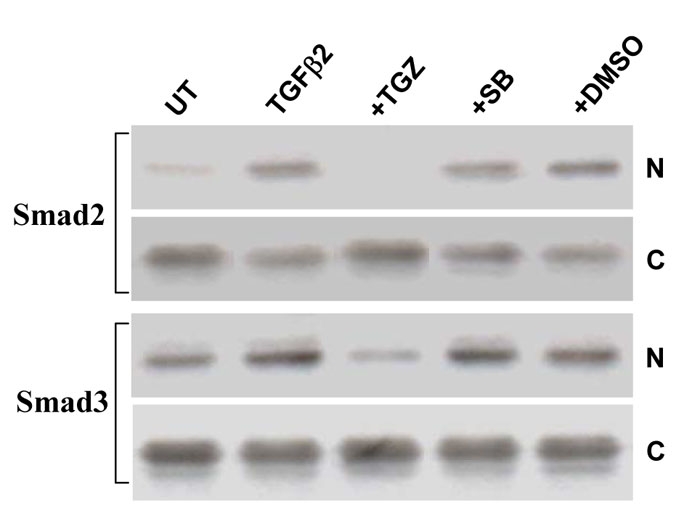
TGFβ2-induced translocation of Smads2/3 from the cytosolic to nucleus fractions is inhibited by TGZ.****ARPE-19 cells were treated with 4 ng/ml TGFβ2 alone or pretreated with 10 μM TGZ, 10 μM SB203580 or DMSO for 1 h and then stimulated with TGFβ2 for 2 h. Cells were harvested and aliquots containing equal amounts of protein from cytosolic (C) and nucleus (N) fractions were subjected to SDS–PAGE and western blot analysis. The result of one representative assay from two similar independent experiments is shown.

TGFβ binding to its receptor causes phosphorylation of Smad2 and Smad3 to form a hetero-oligomeric complex with Smad4, which then translocates into the nucleus to regulate transcription of target genes [25]. We thus examined if TGZ prevented Smad2 and 3 phosphorylation that could also block cytoplasmic Smad translocation to nucleus. Since TGZ can suppress TGFβ2-induced early (2 h) Smad 2 and 3 phosphorylation, the treated cells were subjected to subcellular fractionation and the TGZ effect was examined by western blotting. As shown in Figure 10, as predicted, TGZ prevented TGFβ2-induced Smad2 and Smad3 nuclear translocation (compare the nuclear fractions of TGFβ2 and TGFβ2+TGZ). DMSO or SB203580 pretreatment had no effect.

## Discussion

Presently, we investigated the TGZ effect on TGFβ2-stimulated responses in ARPE-19 cells. Our results reveal that TGZ pretreatment prevents TGFβ2-induced collagen type I and fibronectin expression at both the mRNA and protein levels. TGZ pretreatment also suppresses TGFβ2-induced cell migration. Moreover, we demonstrate that TGZ inhibits TGFβ2-induced phosphorylation of Smad2 and Smad3 and their subsequent nuclear translocation in a time-dependent manner. However, TGZ has no influence on TGFβ2-induced p38 MAPK phosphorylation. Since TGFβ2-induced fibrogenesis of RPE cells is involved in both Smad and p38 MAPK signaling, we propose that the TGZ inhibitory effect on TGFβ2-stimulated ARPE-19 cells is mediated through inhibition of Smad signaling.

PPARγ ligands can protect against several insults to eyes. For example, TGZ can protect against glutamate insult in retinal ganglion cells [26], inhibit vascular endothelial growth factor-induced tube formation of bovine choroidal endothelial cells, and suppress laser photocoagulation-induced choroidal neovascularization lesions in rat and monkey eyes [27]. Our results demonstrate that TGZ can inhibit certain TGFβ2 stimulation in ARPE-19 cells; it may have an additional beneficial effect on preventing the resulting insults of TGFβ2-stimulated RPE cells to the retina.

Human RPE cells express both PPARγ1 and its heterodimeric partner RXRα [27]. Consistent with the findings, we also found PPARγ protein expressed in ARPE-19 cells by immunoblotting (Figures 7A). In the present study, we show that TGZ inhibition of TGFβ2-stimulated responses in ARPE-19 cells is likely to be independent of the PPARγ receptor. This suggestion is supported by the observation that there was no change in the PPARγ protein levels with exposure to TGZ in the presence or absence of TGFβ2 (Figures 7A) and pretreatment for 2 h with 1–20 μM GW9662 (a selective PPARγ antagonist) fails to reverse the inhibition of TGFβ2-induced migration and fibronectin production (Figures 7).

Smad2 and Smad3 are major signaling molecules downstream of TGFβ cell surface receptors concerned with the activation of TGFβ gene targets. Thus, they have been proposed as being a key therapeutic target in the treatment of fibrosis disorders in the eye [6]. Our study demonstrates that TGZ can inhibit TGFβ2-induced early (2 h) Smad2 and 3 and late (24 h) Smad3 phosphorylation in ARPE-19 cells (Figure 9). During the preparation of this our manuscript, a study was published that supports our finding that a PPARγ agonist, GW7845, can suppress TGFβ1-mediated collagen production of HSC via the inhibition of Smad3 phosphorylation [28]. Therefore, it is of interest to study whether TGZ and GW7845 operate through a similar molecular mechanism to inhibit Smad3 phosphorylation.

TGFβ2 also transiently induces p38 MAPK activation (Figure 8A) and p38 MAPK signaling has been proposed, at least in part, in the upregulation of Smad2 and Smad3 activity [13]. Inhibition of p38 MAPK activity also causes suppression of TGFβ2-stimulated responses in ARPE-19 cells [12,13]. Interestingly, both presently and in a previous report [12], a p38 MAPK inhibitor (SB203580) only partially inhibits TGFβ2-induced collagen type I expression (Figures 2 and 3, TGFβ2+SB compared with TGFβ2), suggesting not all Smad activities are controlled by p38 MAPK in ARPE-19 cells. TGZ combining with the p38 MAPK inhibitor may provide an inhibition similar to either treatment alone at a high dosage, leading to decrease side-effects in vivo.

The underlying mechanism of TGZ suppression of TGFβ2-induced Smad2 and 3 phosphorylation remains unclear. The levels of Smad phosphorylation are induced by binding to TGFβ receptors (activin-receptor-like kinases; ALKs) [25]. Decrease of Smad2 and Smad3 phosphorylation levels by TGZ may be through interference with the TGFβ2-ALKs interaction or suppression of the intrinsic serine/threonine kinase activity of ALKs or an increase in an association between ALKs and Smad7 (an inhibitory Smad) [6] or induction of Smad7 expression [29]. In addition, phosphorylation of Smad2 and Smad3 is regulated by a phosphatase, PPM1A, that directly dephosphorylates the Smad species to limit their activation [30]. These are candidate mediators of the TGZ effect. The mechanisms involved in the inhibition of Smad2 and Smad3 phosphorylation by TGZ will be important to identify in future research. In this regard, further study of the TGZ inhibitory mechanism may helpful to explain why TGZ inhibits TGFβ2-induced C-terminal phosphorylation of Smad3 more effectively than Smad2.

In summary, TGZ pretreatment can suppress certain TGFβ2-stimulated responses in ARPE-19 cells including collagen type I and fibronectin production, cell migration, and the phosphorylation of Smad2 and Smad3. This finding may contribute in part to therapeutic solutions for TGFβ-Smads2/3 pathway-mediated tissue fibrosis.
